# Global proteomic characterization of microdissected estrogen receptor positive breast tumors

**DOI:** 10.1016/j.dib.2015.09.034

**Published:** 2015-10-08

**Authors:** Tommaso De Marchi, Ning Qing Liu, Christoph Sting, Marcel Smid, Mila Tjoa, René B.H. Braakman, Theo M. Luider, John A. Foekens, John W.M. Martens, Arzu Umar

**Affiliations:** a Department of Medical Oncology, Erasmus MC Cancer Institute, University Medical Center, Rotterdam, The Netherlands; b Department of Neurology, Erasmus MC, University Medical Center, Rotterdam, The Netherlands; c Cancer Genomics Center Netherlands, Amsterdam, The Netherlands

## Abstract

We here describe two proteomic datasets deposited in ProteomeXchange via PRIDE partner repository [1] with dataset identifiers PXD000484 (defined as “training”) and PXD000485 (defined as “test”) that have been used for the development of a tamoxifen outcome predictive signature [2]. Both datasets comprised 56 fresh frozen estrogen receptor (ER) positive primary breast tumor specimens derived from patients who received tamoxifen as first line therapy for recurrent disease. Patient groups were defined based on time to progression (TTP) after start of tamoxifen therapy (6 months cutoff): 32 good and 24 poor treatment outcome patients were comprised in the training set, respectively. The test set included 41 good and 15 poor treatment outcome patients. All specimens were subjected to laser capture microdissection (LCM) to enrich for epithelial tumor cells prior to high resolution mass spectrometric (MS) analysis. Protein identification and label-free quantification (LFQ) were performed with MaxQuant software package [3]. A total of 3109 and 4061 proteins were identified and quantified in the training and test set, respectively. We here present the first public proteomic dataset analyzing ER positive recurrent breast cancer by LCM coupled to high resolution MS.

## Specifications table

**Table:** 

Subject area	*Biology*
More specific subject area	*Clinical Proteomics*
Type of data	1. *RAW MS Orbitrap XL data* 2. *MaxQuant “Protein groups.txt” output*
How data was acquired	*LTQ Orbitrap XL MS interfaced with a reverse phase column (PepMap C18, 75* *µm ID x 50* *cm, 3* *µm particle size, 100* *Å pore size).*
Data format	*RAW;.txt*
Experimental factors	*All ER positive fresh frozen breast cancer tissues were subjected to LCM to enrich for epithelial tumor cells prior to protein digestion, which enabled analysis of highly pure subpopulations of breast cancer cells.*
Experimental features	1. *Cryo-sectioning of breast cancer tissues and collection on polyethylene–naphtalate coated slides* 2. *Hematoxylin staining and LCM-enrichment* 3. *Protein digestion (MS Grade Trypsin)* 4. *LC–MS analysis* 5. *Label-free quantitation (LFQ) by MaxQuant software*
Data source location	*Rotterdam, The Netherlands*
Data accessibility	*PXD000484:*http://proteomecentral.proteomexchange.org/cgi/GetDataset?ID=PXD000484
*PXD000485:*http://proteomecentral.proteomexchange.org/cgi/GetDataset?ID=PXD000485

## Value of the data

• First public proteomics datasets of LCM derived ER positive primary tumor cells analyzed by high resolution MS.
• Characterization of proteomic changes related to resistance to first line tamoxifen therapy.
• Quantification of 3109 and 4061 unique proteins in training and test sets, respectively.

## 1 Materials and methods

### 1.1 Sample sets

We collected a total of 112 fresh frozen ER positive breast cancer tissues that displayed a minimum ( ≥) of 40% tumor area and that were collected from patients who received tamoxifen therapy for recurrent disease and no adjuvant hormonal therapy after resection of the primary tumor. Patient groups were defined based on outcome to tamoxifen therapy for recurrent disease: patients who manifested progression of disease within (≤) 6 months after start of therapy were defined as manifesting poor outcome, while the good outcome group comprised patients with disease progression after (>) 6 months. Patient samples in the training set (PXD000484) were collected from Erasmus Medical Center (*n*=56; 32×good, 24×poor), while the test set (PXD000485) comprised tumors collected from the Netherlands Cancer Institute – Antoni van Leeuwenhoek hospital (*n*=41) and Radboud University Medical Center (*n*=15), which comprised 41 good and 15 poor outcome patients, respectively, as previously reported (Ref. [2]). Clinical information for every patient in the training an test sets are reported in Tables S1 and S2, respectively.

### 1.2 Sample preparation

Breast cancer tissue samples were processed according to our previously reported tissue proteomic workflow [4], [5]. Frozen tissue specimens were cut into 8 µm cryo-sections, collected on polyethylene naphtalate coated glass slides, and stained with hematoxylin. From each sample, around 4000 epithelial tumor cells were collected through LCM (corresponding to an area of ~500,000 µm^2^) and suspended into 20 µl of 0.1% w/v Rapigest/50 mM ammonium bicarbonate solution.

### 1.3 Protein digestion

LCM collected tissues were lysed through sonication at 70% amplitude. Proteins were denatured at 95 °C, reduced with a 100 µM dithiothreithol solution, and alkylated with a 300 mM iodoacetamide solution. MS grade trypsin was added in a 1:4 enzyme–protein ratio and incubated for 4 h at 37 °C. Digested samples were then acidified with trifluoroacetic acid and spun down at 14,000 RPM. Supernatants were collected and transferred to HPLC vials for further MS measurement.

### 1.4 High resolution MS analysis

MS measurements were performed as previously described with on an LTQ Orbitrap XL interfaced with a nano liquid chromatography system (Ultimate 3000, Dionex, Amsterdam, The Netherlands) [2], [5], [6]. Digested proteins were separated on a reverse phase analytical column (PepMap C18, 75 μm ID×50 cm, 3 μm particle size and 100 Å pore size) in a 3 h gradient: 2 h 0–25% mobile phase B (80% acetonitrile and 0.08% formic acid), and 1 h 25–50% mobile phases B and A (2% acetonitrile and 0.1% formic acid in purified water). The top 5 most intense peaks in full scan (from 400 to 1800 Th) were fragmented by collision induced dissociation.

### 1.5 Protein identification and quantitation

Orbitrap.RAW files were analyzed by MaxQuant (v1.2.2.5), using Andromeda for peptide search [3], [7]. UniProt-SwissProt human canonical database (version 2012-09, human canonical proteome; 20,243 identifiers) was used as reference database. For identification, peptide length was set to 7 aminoacids, match between runs was enabled and settings were kept as default. All other settings were set as default. “Protein groups.txt” files were uploaded in ProteomeXchange along with Orbitrap.RAW files.

## Financial support

This study was supported by the Dutch Cancer Society (KWF), EMCR2009-4319 and the CTMM-Breast Care project 030-104-06.
